# Lower-Limb Kinematic Change during Pelvis Anterior and Posterior Tilt in Double-Limb Support in Healthy Subjects with Knee Malalignment

**DOI:** 10.3390/ijerph19159164

**Published:** 2022-07-27

**Authors:** So Hyun Park, Min Sik Yong, Hae Yong Lee

**Affiliations:** Department of Physical Therapy, Youngsan University, 288, Junam-ro, Yangsan-si 50510, Gyeongnam, Korea; ptpsh@ysu.ac.kr

**Keywords:** Q-angle, lower limb, pelvic tilt

## Abstract

This study aimed to investigate lower-limb kinematic changes during pelvic tilting in participants with knee malalignment. To define participants with lower-limb malalignment, the quadriceps angle (Q-angle) was used in this study. The sample population was divided into two groups in accordance with the Q-angle: the experimental group (ABQ) consisted of participants with an abnormal Q-angle greater than the normal range, and the control group (CON) consisted of participants with a normal Q-angle. All participants performed anterior and posterior pelvic tilts in double-limb support. Kinematic change in the lower limb was evaluated using a three-dimensional motion analysis system (Motion Analysis, Santa Rosa, CA, USA). The horizontal plane hip angle in the ABQ was significantly different compared with that in the CON in all positions (*p* < 0.05), and no significant difference was observed in the other lower-limb kinematic variables (*p* > 0.05). A significant correlation was identified only between the Q-angle and horizontal plane hip angle in all positions. Based on the results, the Q-angle was strongly related to the thigh, although it may not be related to malalignment with other segments during double-limb support.

## 1. Introduction

Quadriceps angle (Q-angle) is widely used as a general measurement to evaluate knee alignment in clinical practice. This angle is formed by the intersection of the line from the anterior superior iliac spine (ASIS) to the midpoint of the patella and the line from the midpoint of the patella to the tibial tuberosity [1,2]. Q-angle refers to the magnitude of the lateral movement of the patella generated by the quadriceps contraction [3], thus it is an important indicator for alignment of the knee joint [3].

A Q-angle is considered normal if it is between 12° and 20° [4]; men tends to be at the low end of this range while women tend to have higher values. Q-angle is usually regarded as excessive when an angle is >20° [3,5]. An increase in Q-angle can alter the biomechanics of the knee and lead to lateral patellar dislocation or increased lateral patellofemoral contact pressures, which can further cause valgus knee, potential sports injuries, and pathological conditions such as patellofemoral pain syndrome or knee instability [6,7,8,9].

Excessive Q-angle can appear in healthy young people without knee symptoms. In these situations, biomechanical analysis under various conditions is required to assess the ability of this condition to cause future pathological conditions [10].

Due to compensatory posture, knee malalignment affects not only the lower extremities but also the alignment of the pelvis [11,12]. Conversely, the position of the pelvis plays a role in controlling the alignment of the lower extremities [13,14]. Therefore, closely examining the effect of changes in pelvic posture on changes in all directions of the hip, knee, and ankle joints is necessary for subjects with knee malalignment. However, three-dimensional data on each joint of the lower limb during manipulation of the pelvis for people with malalignment of the knee joint remains limited.

Thus, we posit that when the pelvic posture changes, the kinematics of the lower limb also changes, and this phenomenon is more exaggerated in patients with knee malalignment. To define patients with knee malalignment, the Q-angle was used in this study [1,2].

Here, we evaluate the lower-limb kinematic change according to the anterior and posterior tilt of the pelvis in a static double-limb support posture support in healthy subjects with knee malalignment using a three-dimensional motion capture analysis system. For this study, the Q-angle was used to define subject with knee malalignment.

## 2. Methods

### 2.1. Participants

**Figure 1:**
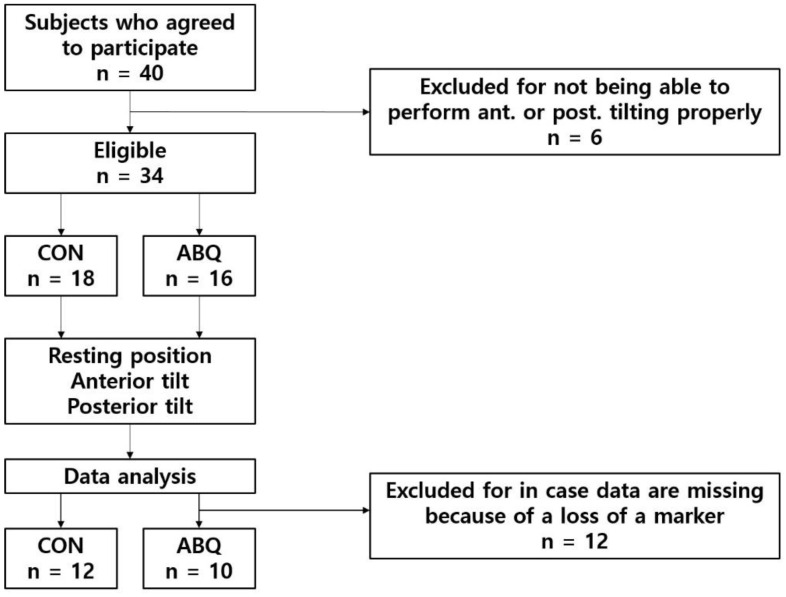
Flow chart of recruitment.

This prospective, cross-over study was conducted at Youngsan University, Korea. The participants were recruited through an advertisement on the University notice board. Among the potential participants aged ≥18 years who were capable of normal gait and volunteered to participate in the study, 40 individuals meeting the following criteria were selected (Table 1). The inclusion criteria were as follows: no pathological abnormality in the knee joint, no history of lower extremity trauma or surgery, no pathological development in the lower-limb bones, and no muscle convulsion or stiffness. The exclusion criteria were as follows: discomfort due to lower extremity pain upon daily activity, moderate pain with a visual analog scale score of above 5 after performing squats ≥20 times, inability to complete a 100 m gait, presence of a tumor, and pregnancy (Figure 1).

Each participant provided informed consent before the study initiation. The study adhered to the principles of the Declaration of Helsinki and was approved by the local and central ethics committees of Youngsan University (YSUIRB-201506-HR-001-02).

The Q-angle was measured, with the participants in the supine position, by an experienced physical therapist. The angle formed by the intersection of the lines from the anterior superior iliac spine (ASIS) to the midpoint of the patella and from the midpoint of the patella to the tibial tuberosity is the Q-angle [1]. The normal Q-angle score is between 12° and 20° [4,5]. All participants were divided into two groups according to their Q-angle: the experimental group (ABQ) consisting of participants with an abnormal Q-angle greater than the normal range, and the control group (CON) consisting of participants with a normal Q-angle.

### 2.2. Determination of the Sample Size

For sample size determination, the Q-angle became the primary end-point in this study. The significance level (α) = 0.05, power (1 − β) = 0.85, effect size = 1, and a two-tail test were applied. At least 19 participants were required for each group, and thus, a total of 40 participants were enrolled to cope with potential losses.

### 2.3. Protocol

**Figure 2:**
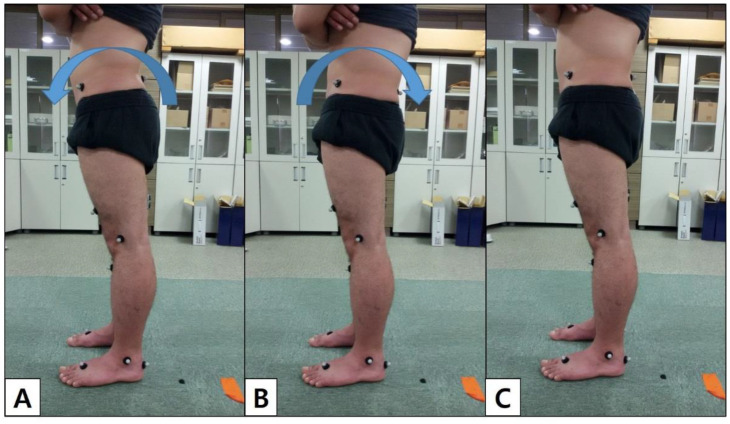
Illustration showing the double-leg support with pelvis anterior (**A**), posterior tilt posture (**B**), and resting posture (**C**) in lateral view.

Participants were instructed by one physical therapist to practice an anterior and posterior pelvic tilt in double-limb support. They were then provided with a 20 min orientation, followed by a 10 min rest before the experiment. The physical therapist commanded that the participants perform a comfortable standing posture with a cross-armed position, keeping their feet and legs shoulder-width apart for double-limb support for the resting position. Additionally, the pelvis must be turned forward for anterior pelvic tilt or backward for posterior pelvic tilt as far as possible, and slightly natural knee flexion was permitted in each position (Figure 2). At this time, 6 subjects whose position could not be maintained constantly because they did not understand the performing anterior and posterior pelvic tilt were excluded.

All measurements were taken in triplicate. The measurements were taken during the last 10 s as the participant halted to maintain the posture.

### 2.4. Data Analysis

A three-dimensional motion analysis was performed using six cameras with a Falcon system (Motion Analysis, Santa Rosa, CA, USA). The joint angle is calculated as the angular value between the parent and child segments. The camera was set to acquire 60 frames per second at a sampling rate of 60 Hz. Reflective spherical markers (14 mm) were taped onto the skin of the lower extremities using a Helen Hayes marker set. Fifteen reflective spherical markers (Helen Hayes marker set) were placed on the left and right ASIS, midthighs, midshanks, lateral femoral epicondyles, lateral malleoli, second metatarsals, calcaneus, and sacrum. Four additional spherical markers were taped to the medial epicondyles of the femur and medial malleoli for calibration of the standing position (static data). The markers were placed on all participants by the same individual.

All participants performed anterior and posterior pelvic tilts in double-limb support. Three trials for each position (anterior pelvic tilt, posterior pelvic tilt, and resting pelvic position) were recorded for further analysis using the Cortex 3.0 software program (Motion Analysis Corp., Rohnert Park, CA, USA), and three-dimensional joint angles for the hip, knee, and ankle were determined. Positive values represent extension, adduction, and external rotation in the hip joint; flexion, adduction, and external rotation in the knee joint; and plantar flexion, inversion, and external rotation in the ankle joint.

During data processing, missing data occurred in 12 subjects. For accurate analysis, if occlusion of the marker occurred in one joint of any motion, it was considered as missing data. Because these data values existed as outliers, they had a great influence on the mean. Thus, 12 participants were excluded from subsequent analyses, which left the data of 22 of 40 participants in the final analyses in this study.

### 2.5. Statistical Analysis

The independent t-test was used to examine the effect of the Q-angle on the changes in each joint (hip, knee, and ankle) between pelvic motions, and Pearson’s correlation coefficients were used to determine the relationship between the Q-angle and lower-limb kinematic variables. Statistical analyses were performed using SPSS version 25.0 (IBM Corp., Armonk, NY, USA), and statistical significance was set at *p* < 0.05.

## 3. Results

Patient demographic data are summarized in Table 1. Regarding the effects of the Q-angle on lower-limb kinematic variables in each position, the horizontal plane hip angle in the ABQ was significantly different compared with that in the CON in all positions (*p* < 0.05). No significant difference was observed in the other lower-limb kinematic variables (*p* > 0.05) (Table 2).

Regarding the correlations between the Q-angle and lower-limb kinematic variables in each position, a significant correlation was identified only between the Q-angle and the horizontal plane hip angle in all positions (*r* = −0.547, *p* = 0.008; *r* = −0.482, *p* = 0.023; and *r* = −0.629, *p* = 0.002, respectively). No correlation was observed between the Q-angle and the other lower-limb kinematic variables (Table 3).

## 4. Discussion

In this study, an increase in the internal rotation of the hip joint in the Q-angle group was observed during resting standing, indicating a correlation between the Q-angle and hip rotation. Many studies have reported the Q-angle as an independent risk factor for increasing knee pain, such as patellofemoral pain syndrome and ankle sprain [15,16,17]. The larger the Q-angle, the greater the lateral bowstringing force, which can reduce the contact area and increase the possibility of patellar dislocation [18,19]. Moreover, larger Q-angles can lead to instability and imbalance in the pressure on joints [20]. The Q-angle has been proposed to measure a combination of pelvic, hip, tibial, patellar, and foot positions [21,22]. In particular, the Q-angle may increase with excessive anterior pelvic tilt, femoral anteversion, knee valgus, and tibial external rotation [22].

However, in this study, only increased internal rotation of the hip joints was observed with the increase in Q-angle, and no significant difference in the kinematic knee and ankle joints was identified.

Additionally, our results reveal that during anterior and posterior tilting, the internal rotation of the hip joint increased in the Q-angle group. The relationship between pelvic tilt change and the lower limb has been verified previously [23,24,25,26]. However, the relationship between pelvic tilt and lower-limb motion along the Q-angle is less well documented. Although the anterior pelvic tilt and internal rotation of the hip joint have a strong relationship, a weak direct relationship exists between pelvic alignment and the foot [16]. In fact, the foot and pelvic alignment relationship, while standing directly on the floor, has been reported to be the worst [14,16]. Khamis et al. investigated the relationship between anterior pelvic tilt and thigh internal rotation, and only the shank significantly affected pelvic alignment, acting as a mediator between the foot and thigh, with the thigh having a crude significant effect on the pelvis [14,16]. However, our results reveal no relationship between pelvic tilt and the shank. Internal tibial rotation was coupled with external femoral rotation, and external tibial rotation with internal femoral rotation. However, unidirectional coupling occurs between the two segments in the standing position [16]. In this study, the participants were positioned standing shoulder-width apart with both feet parallel. Nguyen et al. investigated the relationship between the Q-angle, increased tibiofemoral angle, and increased femoral anteversion and discovered no significant relationships between the Q-angle and other parameters (pelvic angle, genu recurvatum, tibial torsion, navicular drop, and femur to tibia length ratio) [27]. Specifically, the tibiofemoral angle and femoral anteversion have the strongest association with a greater Q-angle [27]. Femoral anteversion indicates medial torsion of the femur when the femoral neck projects forward to the femoral condyles [28]. Excessive femoral anteversion places the femur in a more medial rotated position, potentially resulting in a medial displacement of the patella. Changes in the tibiofemoral angle may have a substantially greater impact on the magnitude of the Q-angle than femoral anteversion [27]. A previous study has also reported that an anterior pelvic tilt results in the acetabulum shifting backward, causing the femur to rotate internally on the pelvis [16]. Thus, in the standing position, the increased Q-angle is related to internal rotation of the thigh during pelvic tilt. The most important factor in increased Q-angle and pelvic tilt in the standing posture may be considered the rotation of the thigh.

The anterior pelvic tilt increases the lumbar lordosis, and the posterior pelvic tilt decreases the lumbar lordosis (contradirectional lumbopelvic rhythm) [20,29]. Active pelvic tilt is caused by contraction of the hip and lumbar muscles [14]. The pelvic position is highly related to the lumbar position [29]. Both hip joints were internally rotated, and the torque acting on the vertical axis of the pelvic girdle was eliminated (correlation between Q-angle and isokinetic knee strength). Therefore, a larger Q-angle may have internal rotation of the hip joint, limiting the function of the hip muscles and having a greater effect on lumbar and abdominal muscle contraction.

Consequently, even if the Q-angle is large, it may not be related to lower-extremity malalignment when anterior and posterior tilting is performed. During weight-bearing activities, the internal rotation of the hip can be caused by reduced muscle strength or neuromuscular control of the hip joint lateral rotation muscles, tightness of the internal rotator of the hip joint, excessive anteversion, or excessive shank lateral torsion. The weakening of the postural phenomena of the hip lateral rotator and abductor muscles is also common in healthy women [30,31] and is an important and widespread factor that increases the risk of recurrent dislocation and development of pain in the patellofemoral joint [32,33]. A greater Q-angle is associated with decreased isokinetic knee strength, power output, and torque angles [15]. The increased VM (vastus medialis) muscle activity due to squats can reduce Q-angle [34], and the minimized change in Q-angle can optimize the biomechanics of cycling [35]. Identifying the muscle weakness caused by the increase in Q-angle, the change in peak torque caused by the change in quadriceps vector direction, and other factors may be more important than the Q-angle as a malalignment factor of the lower extremity.

Although pelvic tilt has a strong relationship with the internal rotation of the thigh or shank, other factors may have a greater influence on the knee and ankle joints than on the thigh. As previously studies, both increased anterior pelvic tilt and posterior pelvic tilt would result in rotational changes in the femur and tibia [21,22,36,37], but displacing the patella medially and the tibial tuberosity laterally, which may not be sufficient to alter the landmarks. It is needed to figure out what factors could have an influence on the knee and ankle joints to identify indiciduals at risk of knee and ankle injuries because the measurement of the Q-angle alone may not be sufficient..

## 5. Limitations

This study had several limitations that warrant consideration. First, this study involved a relatively small sample size. Second, the effect of the pelvic tilt on the lumbar spine was not assessed.

## 6. Conclusions

The Q-angle and pelvic tilting was strongly related to the rotation of the thigh, but it was not related to malalignment with other segments during double-limb support. The most important factor in increased Q-angle and pelvic tilt in the standing posture may be considered the rotation of the thigh. The increase in internal rotation of the thigh should be considered for pelvic tilt, and the measurement of the Q-angle alone may not be sufficient to identify individuals at risk of injuries. In addition, the Q-angle measurement should be performed with measurement of the pelvic tilt.

## Figures and Tables

**Table 1:** General characteristics of the participants.

Variables	CON (*n* = 12)	ABQ (*n* = 10)
Gender(male/female)	7/5	3/7
Age (years)	23.08 ± 1.72	22.50 ± 0.97
Height (cm)	168.33 ± 7.43	164.30 ± 10.02
Weight (kg)	66.58 ± 10.95	57.50 ± 12.56
Q-angle (°)	17.08 ± 2.50	27.00 ± 2.98

All values are mean ± standard deviation.

**Table 2:** Effects of Q-angle on lower-limb kinematic variables in each position.

Pelvic	Joint	Kinematics	ABQ	CON	t	*p*
Resting Position		Flex/Ext	−4.14 ± 4.53	−4.57 ± 5.16	−0.21	0.83
	Hip (°)	Add/Abd	−2.91 ± 2.47	−3.28 ± 3.57	−0.28	0.77
		Int/Ext	−5.36 ± 4.97	−0.54 ± 4.61	2.33	0.03 *
		Flex/Ext	0.63 ± 6.41	0.99 ± 3.99	0.16	0.87
	Knee (°)	Add/Abd	−1.26 ± 2.41	−2.10 ± 3.70	−0.64	0.53
		Int/Ext	10.15 ± 7.04	5.48 ± 10.02	−1.28	0.21
		Dorsi/Plantar	10.89 ± 3.81	10.55 ± 3.25	−0.22	0.82
	Ankle (°)	Inv/Ev	−1.76 ± 5.56	−4.11 ± 5.73	−0.97	0.34
		Add/Abd	14.62 ± 3.10	11.95 ± 4.37	−1.67	0.11
Anterior Tilt		Flex/Ext	−20.27 ± 7.73	−17.74 ± 6.59	0.81	0.42
	Hip (°)	Add/Abd	−3.46 ± 2.55	−4.47 ± 2.41	−0.94	0.35
		Int/Ext	−6.10 ± 5.09	−1.17 ± 5.26	2.22	0.03 *
		Flex/Ext	−1.31 ± 8.59	−6.38 ± 2.94	−1.91	0.07
	Knee (°)	Add/Abd	−1.02 ± 2.47	−1.80 ± 4.18	−0.54	0.59
		Int/Ext	11.10 ± 7.31	7.90 ± 9.63	−0.88	0.38
		Dorsi/Plantar	13.77 ± 3.77	13.32 ± 3.23	−0.29	0.77
	Ankle (°)	Inv/Ev	−0.56 ± 5.14	−2.89 ± 5.63	−1.01	0.32
		Add/Abd	14.29 ± 3.52	11.08 ± 4.46	−1.88	0.07
Posterior Tilt		Flex/Ext	4.02 ± 3.41	4.37 ± 4.92	0.19	0.84
	Hip (°)	Add/Abd	−3.60 ± 2.66	−2.78 ± 2.50	0.73	0.46
		Int/Ext	−1.63 ± 8.55	6.12 ± 2.23	3.02	0.01 *
		Flex/Ext	7.38 ± 8.42	8.54 ± 4.49	0.41	0.68
	Knee (°)	Add/Abd	−0.70 ± 2.72	−1.68 ± 3.67	−0.71	0.48
		Int/Ext	7.93 ± 6.71	5.77 ± 9.83	−0.61	0.54
		Dorsi/Plantar	7.57 ± 3.87	5.88 ± 3.32	−1.08	0.29
	Ankle (°)	Inv/Ev	−1.98 ± 5.88	−1.91 ± 6.48	0.02	0.97
		Add/Abd	15.66 ± 3.36	13.28 ± 4.23	−1.53	0.14

All values are mean ± standard deviation. Positive values represent extension, adduction, external rotation in the hip joint; flexion, adduction, external rotation in the knee joint; and plantar flexion, inversion, external rotation in the ankle joint. Abbreviation: Flex, flexion; Ext, extension; Add, adduction; Abd, abduction; int, internal rotation; Ext, external rotation; Dorsi, dorsiflexion; Plantar, plantar flexion; Inv, inversion; and Ev, eversion. * *p* < 0.05.

**Table 3:** Pearson correlation between Q-angle and lower-limb kinematic variables in each position.

Pelvic	Joint	Kinematics	*R*	*p*
Resting Position		Flex/Ext	0.203	0.364
	Hip	Add/Abd	0.135	0.549
		Int/Ext	−0.547	0.008 **
		Flex/Ext	−0.166	0.461
	Knee	Add/Abd	0.156	0.489
		Int/Ext	0.385	0.077
		Dorsi/Plantar	0.007	0.976
	Ankle	Inv/Ev	0.159	0.479
		Add/Abd	0.241	0.279
Anterior Tilt		Flex/Ext	−0.011	0.961
	Hip	Add/Abd	0.307	0.165
		Int/Ext	−0.482	0.023 *
		Flex/Ext	0.122	0.587
	Knee	Add/Abd	0.104	0.645
		Int/Ext	0.304	0.169
		Dorsi/Plantar	0.106	0.638
	Ankle	Inv/Ev	0.151	0.502
		Add/Abd	0.237	0.287
Posterior Tilt		Flex/Ext	−0.179	0.425
	Hip	Add/Abd	0.073	0.747
		Int/Ext	−0.629	0.002 **
		Flex/Ext	−0.353	0.107
	Knee	Add/Abd	0.184	0.411
		Int/Ext	0.315	0.153
		Dorsi/Plantar	0.328	0.136
	Ankle	Inv/Ev	−0.102	0.653
		Add/Abd	0.149	0.508

All values are mean ± standard deviation. Abbreviation: Flex, flexion; Ext, extension; Add, adduction; Abd, abduction; int, internal rotation; Ext, external rotation; Dorsi, dorsiflexion; Plantar, plantar flexion; Inv, inversion; and Ev, eversion. * *p* < 0.05, ** *p* < 0.01.

## Data Availability

The data presented in this study are available in the article.
